# Simultaneous Measurements of Refractive Index and Methane Concentration through Electromagnetic Fano Resonance Coupling in All-Dielectric Metasurface

**DOI:** 10.3390/s21113612

**Published:** 2021-05-22

**Authors:** Hai Liu, Xu Zhang, Benlei Zhao, Bo Wu, Hancheng Zhang, Shoufeng Tang

**Affiliations:** 1 Engineering Research Center of Intelligent Control for Underground Space Ministry of Education, China University of Mining and Technology, Xuzhou 221116, China; sieeoe@cumt.edu.cn; 2 School of Information and Control Engineering, China University of Mining and Technology, Xuzhou 221116, China; TS19060014A31@cumt.edu.cn (X.Z.); TS19060015A31@cumt.edu.cn (B.Z.); TS19060007A31@cumt.edu.cn (B.W.); TS19060013A31@cumt.edu.cn (H.Z.); 3 Key Laboratory of Gas and Fire Control for Coal Mines, China University of Mining and Technology, Ministry of Education, Xuzhou 221116, China

**Keywords:** Fano resonance, all-dielectric metasurface, dual-parameter measurement, methane sensor

## Abstract

Dual-parameter measurements of refractive index and methane concentration based on electromagnetic Fano resonance are proposed. Two independent Fano resonances can be produced through electric dipole and toroidal dipole resonance in an all-dielectric metasurface separately. The linear relationship between the spectral peak-shifts and the parameters to be measured will be obtained directly. The refractive index (RI) sensitivity and gas sensitivity are 1305.6 nm/refractive index unit (RIU), −0.295 nm/% for one resonance peak (dip1), and 456.6 nm/RIU, −0.61 nm/% for another resonance peak (dip2). Such a metasurface has simpler structure and higher sensitivity, which is beneficial for environmental gas monitoring or multi-parameter measurements.

## 1. Introduction

Fano resonance is caused by the destructive interference between dark mode and bright mode in the near field [1,2]. At the Fano resonance position, the system radiation attenuation can be effectively suppressed. Such a resonance effect will lead to larger field enhancement and a finer spectrum, which is beneficial for achieving greater sensing sensitivity. A metasurface based on Fano resonance has more and more applications due to its unique optical properties, and researchers have proposed many metasurface-based optical devices in different fields, including optical filters [3], optical absorbers [4,5], encoding images [6], and angle sensors [7]. With the rapid development of nanotechnology, different metasurface sensors based on Fano resonance also have been reported, such as refractive index sensors [8,9,10,11,12], biosensors [13,14,15,16,17,18,19,20], and gas sensors [21]. However, quite a lot of metasurface structures are based on a metal layer [22,23,24,25], which would produce higher Joule losses and lower quality factor. All-dielectric materials can solve the above problem because they have much lower Joule losses. Besides this, all-dielectric materials have many other merits, such as low cost, and a simple manufacturing process. In recent years, all-dielectric metasurfaces have attracted many attentions. In 2018, Liu et al. proposed a metasurface based on a silicon split-ring, with only one Fano resonance in the transmission spectrum [12]. In 2019, Yildirim et al. proposed a refractive-index sensor in an all-dielectric metasurface, which was polarization-insensitive and had a high Q-factor [26]. In 2020, Su et al. proposed an all-dielectric metasurface based on asymmetrical elliptical ring-disks, which had a high figure of merit [27]. In 2021, Wang et al. investigated toroidal dipole resonances at terahertz frequencies in an all-dielectric metasurface consisting of an array of high-index tetramer clusters, which achieved an ultra-high sensitivity level of 489 GHz/RIU [28]. It can be seen that all-dielectric devices have excellent performances. Based on the reported research, we propose an all-dielectric metasurface based on Fano resonance. In the transmission spectrum of this metasurface, there exist two uncorrelated Fano resonances that are excited by the electromagnetic field coupling of electric dipole or toroidal dipoles. The designed metasurface has simpler structure, a higher quality factor, and relatively high sensitivity. It can be used for environmental gas monitoring or multi-parameter measurements. Besides this, based on the previous research [29,30,31], it has the potential to be used to integrate with microfluidic channels for sensing of biological analytes.

## 2. Design and Simulation

Based on the previous research [32,33,34,35], the far-field scattering intensity of metasurface can be expressed by Equation (1), in which $\vec{P}$ is electric dipole moment; $\vec{M}$ is magnetic dipole moment; $\vec{T}$ is toroidal dipole moment; $Q_{\alpha ,\beta }^{\left(e\right)}$ is electric quadrupole moment’ and $Q_{\alpha ,\beta }^{\left(m\right)}$ is magnetic quadrupole; the vector $\vec{j}$ is the current density, respectively; *c* is the speed of light; and ω is the frequency of light.

(1) $$I=\frac{2\omega^{4}}{3c^{3}}|\vec{P}{|}^{2}+\frac{2\omega^{4}}{3c^{3}}|\vec{M}{|}^{2}+\frac{4\omega^{5}}{3c^{4}}(\vec{P}\cdot \vec{T})+\frac{2\omega^{6}}{3c^{5}}|\vec{T}{|}^{2}+\frac{\omega^{6}}{5c^{5}}\sum {\left|Q_{\alpha ,\beta }^{\left(e\right)}\right|}^{2}+\frac{\omega^{6}}{40c^{5}}\sum {\left|Q_{\alpha ,\beta }^{\left(m\right)}\right|}^{2}+O\left(\frac{1}{c^{5}}\right)$$

These multipole oscillations can be expressed by Equations (2)–(6), where $\vec{r}$ is the distance vector, and α,β represents the *x-* and *y*-direction of the coordinate axis.

(2) $$\vec{P}=\frac{1}{i\omega }\int \vec{j}d^{3}r$$

(3) $$\vec{M}=\frac{1}{2c}\int \vec{r}\times \vec{j}d^{3}r$$

(4) $$\vec{T}=\frac{1}{10c}\int \left[(\vec{r}\cdot \vec{j})\vec{r}-2r^{2}\vec{j}\right]d^{3}r$$

(5) $$Q_{\alpha \beta }^{\left(e\right)}=\frac{1}{i2\omega }\int \left[r_{\alpha }{\vec{j}}_{\beta }+{\vec{j}}_{\beta }r_{\alpha }-\frac{2}{3}(\vec{r}\cdot \vec{j})\delta_{\alpha ,\beta }\right]d^{3}r$$

(6) $$Q_{\alpha \beta }^{\left(m\right)}=\frac{1}{3c}\int \left[{\left(\vec{r}\times \vec{j}\right)}_{\alpha }r_{\beta }+\left({\left(\vec{r}\times \vec{j}\right)}_{\beta }r_{\alpha }\right)\right]d^{3}r$$

**Figure 1:**
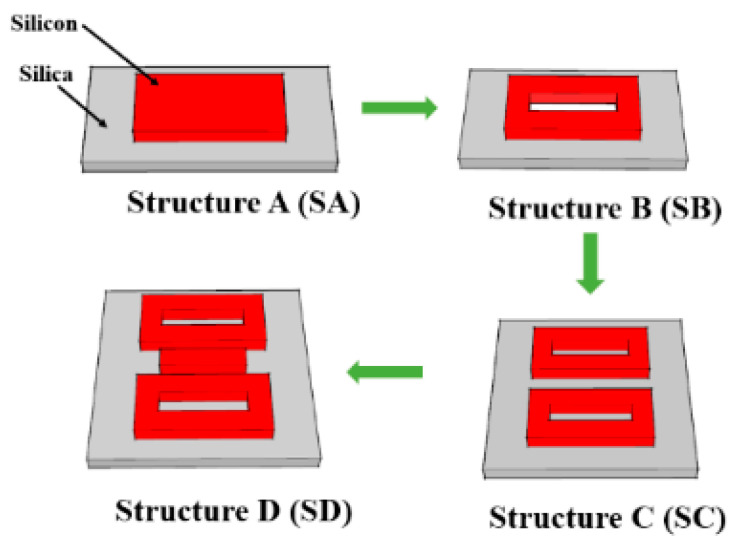
Four types of metasurface to be investigated.

It is worth noting that the resonance characteristics and far-field distributions of metasurfaces are mainly derived from the interference of these multipole oscillations. That is, Fano resonance also can be generated by toroidal dipoles. Therefore, we propose a simple metasurface structure based on electric dipoles and toroidal dipoles together. Obviously, two uncorrelated Fano resonance peaks will appear in the transmission spectra due to the interference of the electric dipoles and toroidal dipoles, respectively. These two different coupling mechanisms provide an effective method for dual-parameter measurement. We designed four metasurface structures having sequential transformation, as is shown in Figure 1. The Structure-A (SA) is composed of a nanobar, and becomes Structure-B (SB) by punching an air hole. The Structure-C (SC) is composed of two toroidal frames, and forms Structure-D (SD) through introducing a nanobar between two frames.

**Figure 2:**
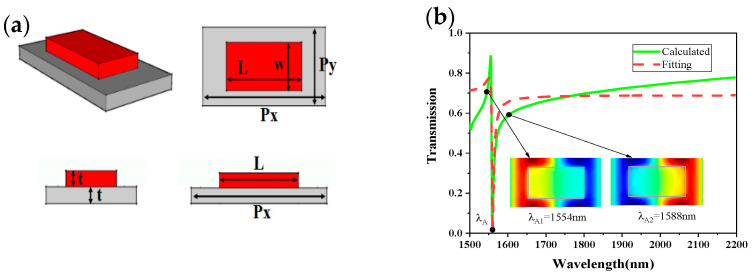
(**a**) The multi-view of Structure-A (SA). (**b**) The transmission spectrum and electric field (E_Z_) distributions of the metasurface SA.

As is well-known, Fano resonance is usually produced through electric dipole resonance, which can be generated by the nanobar. Firstly, we started with SA for the investigation. SA is composed of periodically silicon nanobar on a silica substrate, as is shown in Figure 2a. The thickness of silicon structure and silica substrate are set as *t* = 110 nm, and the length and width of the nanobar are chosen as *L* = 900 nm, ω = 450 nm. The period is selected as $P_{x}$ = 1500 nm and $P_{y}$ = 750 nm, respectively.

The finite-difference time-domain (FDTD) method [36] can solve Maxwell’s differential equations as Equations (7) and (8), where $\vec{E}$ is electric field, $\vec{D}$ is electrical displacement, $\vec{H}$ is magnetic field, $\vec{j}$ is current density, and $\vec{j_{m}}$ is magnetic current density, Through this method, we can calculate the electric field and magnetic field in space.

(7) $$\nabla \times \vec{H}=\frac{\partial \vec{D}}{\partial t}+\vec{j}$$

(8) $$\nabla \times \vec{E}=-\frac{\partial \vec{B}}{\partial t}-\vec{j_{m}}$$

We use the FDTD method to analyze the spectral characteristics of the proposed structure. Perfectly matched layer (PML) boundary conditions are adopted in the z-direction, while periodic boundary conditions are selected in both of *x* and *y*-directions. The incident light is a plane wave, and we define the polarization direction of the electric field with respect to the *x*-axis as *θ*. The material refractive indexes of silica and silicon are reported in Ref. [28]. The transmission spectrum of the metasurface at *θ* = 0° is shown in Figure 2b. It can be seen that a Fano resonance appears at $\lambda_{A}$ = 1559 nm. The spectrum of this proposed metasurface can be fitted by the Fano model [11] shown as Equation (9), where a_1_, a_2_, and *b* are constant numbers, $\omega_{0}$ is the oscillation frequency, and γ is the damping factor. Then, the Q factor of Fano resonance can be calculated by the following Equation (10). The Q-factor of Fano resonance at 1559 nm is about 329.35 ($\omega_{0}=0.91\;\mathrm{eV}$, $\gamma =1.38\times 10^{-3}\mathrm{eV}$).

(9) $$T_{\mathrm{fano}}={\left|a_{1}+{\mathrm{ia}}_{2}+\frac{b}{\omega -\omega 0+i\gamma }\right|}^{2}$$

(10) $$Q=\frac{\omega_{0}}{2\gamma }$$

**Figure 3:**
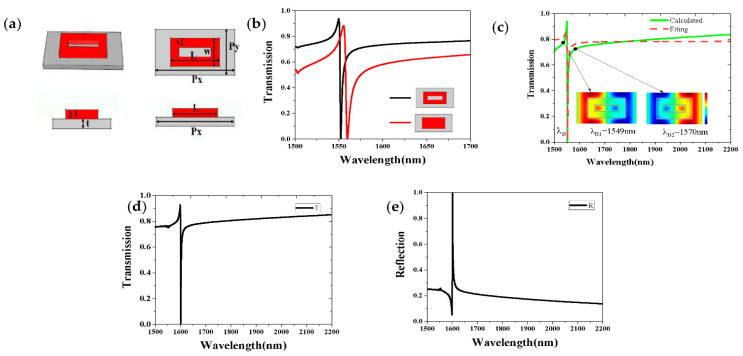
(**a**) The multi-view of structure B(SB) and (**b**) different spectra of SA and SB. (**c**) The transmission spectrum and electric field (E_Z_) distributions of SB. (**d**) The transmission spectrum of SB. (**e**) The reflection spectrum of SB.

In order to explore the Fano resonance mechanism of SA, we calculated the *z*-component of electric field ($E_{Z}$) distributions of the proposed metasurface at the wavelength of $\lambda_{A1}$ = 1554 nm, and $\lambda_{A2}$ = 1588 nm, respectively, as is shown in Figure 2b. From the $E_{Z}$ distribution, there are two antiphase modes that appeared in the metasurface at $\lambda_{A1}$, and $\lambda_{A2}$, and the interference between these two antiphase modes led to a Fano resonance at $\lambda_{A}$ = 1559 nm. It can be seen from the transmission spectrum of SA that the quality factor was not high. The reason may be that the incident light is difficult to pass through the silicon nanorods, causing the incident light (dark mode) to resonate with the electric dipole (bright mode), and interaction is difficult, so the quality factor was not very large. In order to solve this problem, we added a rectangular air-hole on the nanorods to make it easier for the light to pass through the metasurface. As is shown in Figure 3a, a ring-shaped structure SB was formed with a width of 150 nm. We speculated that this new structure could increase the coupling strength and improve the quality factor effectively. Figure 3b verifies the prediction and shows that the transmittance and Q-factor were greatly improved. Especially, the spectrum of the structure SB can be fitted well by Equation (8), and the Q-factor reach up to 741.53 ($\omega_{0}=0.8\;\mathrm{eV}$, $\gamma =0.53\times 10^{-3}\mathrm{eV}$). The fitted curve and the calculated curve have the same resonance wavelength; however, the fitted curve is flatter than the calculated curve. To describe the resonance mechanism of the structure SB clearly, we plotted the z-component of electric field *(*$E_{Z}$*)* distributions in the metasurface at the wavelength of $\lambda_{B1}$ = 1549 nm, and $\lambda_{B2}$ = 1570 nm, respectively, as is shown in Figure 3c. It can be seen that two antiphase modes appeared in the metasurface, and the interference between these two modes led to a sharp Fano resonance appearing at $\lambda_{B}$ = 1552 nm. In addition, it can be found in Figure 3d,e that the electromagnetic field was completely reflected at the Fano-like resonance.

**Figure 4:**
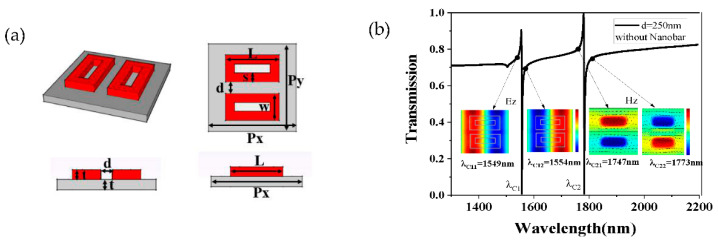
(**a**) The multiview of structure SC and (**b**) the transmission spectrum, electric field (E_z_) distributions, and magnetic field (H_z_) of SC.

Although we have raised the transmittance and Q-factor based on the improved structure SB, there existed only one resonance peak in the transmission spectrum to realize a single-parameter measurement. According to the above analysis results, the toroidal structure can effectively improve the quality factor. Moreover, the toroidal structure can also excite the magnetic dipole mode under the incident light, and these two magnetic dipoles can form a toroidal dipole having different characteristics to a conventional electrical dipole. We speculated that the destructive interference of the toroidal dipole and the incident light in the far field would produce a new Fano resonance. Next, we proceeded to transform SB into SC to achieve a dual-parameter measurement. As is shown in Figure 4a, SC was composed of two toroidal frames with a distance of d = 250 nm, and other parameters were chosen to match those parameters of SB. As is shown in Figure 4b, two resonance peaks appeared in the transmission spectrum at $\lambda_{C1}$ = 1552 nm and $\lambda_{C2}$ = 1756 nm. Obviously, one peak was caused by the interaction between electric dipoles and incident light, and the other was caused by the interaction between toroidal dipole and incident light. To interpret the Fano resonance process of $\lambda_{C1}$, we plotted the *z*-component of electric field ($E_{z}$) distributions at the wavelength of $\lambda_{C11}$ = 1549 nm and $\lambda_{C12}$ = 1554 nm, as is shown in Figure 4b. The interference between these two antiphase modes led to the first resonance peak appearing at $\lambda_{C1}$ = 1552 nm. Meanwhile, we also obtained the *z*-component of magnetic field ($H_{z}$) distributions at the wavelength of $\lambda_{C21}$ = 1749 nm, and $\lambda_{C22}$ = 1787 nm, as is shown in Figure 4b. The black arrows indicate the electric field directions. It can be seen that the magnetic field directions and electric field directions were in the reverse direction at the wavelengths of $\lambda_{C21}$ and $\lambda_{C22}$. The interference between the two antiphase modes resulted in a sharp Fano resonance at $\lambda_{C2}$ = 1756 nm.

**Figure 5:**
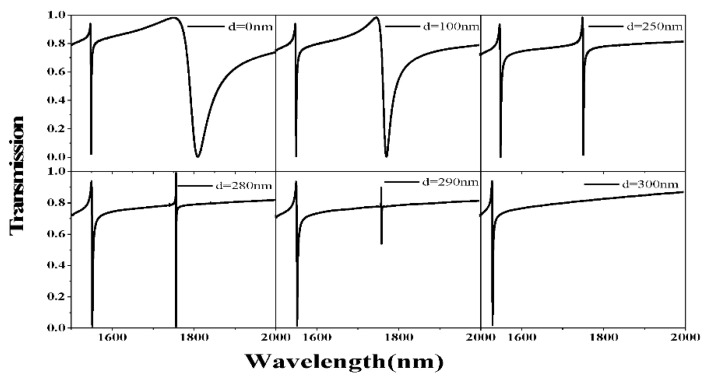
The transmission spectra of the structure SC during the increase of *d* from 0 to 300 nm.

Figure 5 indicates that the first resonance peak always exists when the distance between two toroidal frames *d* increases from 0 nm to 300 nm. However, the second resonance peak will gradually disappear due to the decrease of the interference strength between the toroidal dipole and the incident light. In the case of *d* = 300 nm, there only exists the first resonance peak and the second peak completely disappears. When *d* = 250 nm, both of the two resonance peaks appear in the transmission spectrum. If we choose *d* = 0 nm, there is no gap between the two toroidal frames, and the incident light is difficult to propagate through the metasurface and interfere with the toroidal dipole. As a result, the Q-factor is relatively low when *d* = 0 nm. Since the second Fano resonance may disappear due to the increased distance *d*, we must precisely control the distance between two toroidal frames in the manufacturing process to ensure that two peaks can appear in the transmission spectrum. So, we considered adding a nanobar in the middle of the structure. The nanobar acts as an electric dipole antenna and forms a strong coupling with the incident electric field along the *x*-direction in free space. Furthermore, it interacts with the toroidal resonator through near-field coupling to enhance the magnetic field of the toroidal dipole, and then the disappeared resonance peak can reappear.

**Figure 6:**
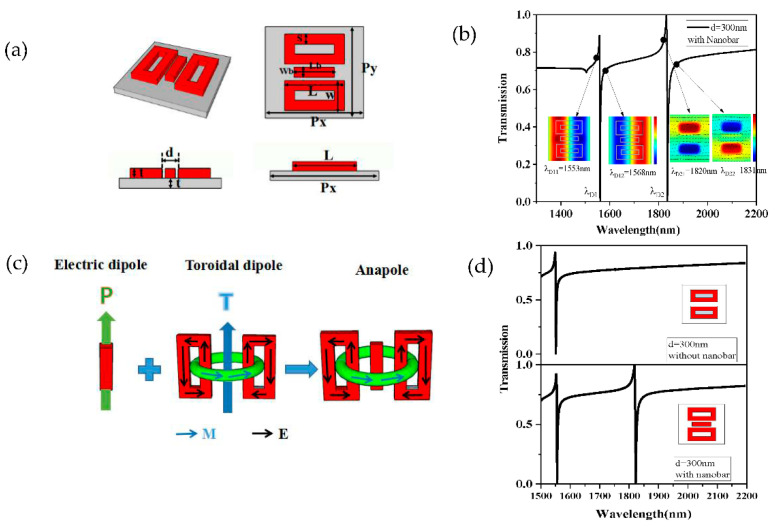
(**a**) The multiview of SD and (**b**) the transmission spectrum, electric field (E_Z_) distributions, and magnetic field (H_Z_) of SD, (**c**) the anapole is produced by the composition of an electric dipole and a toroid dipole. (**d**) The transmission spectra of SC and SD when *d* = 300 nm.

A nanobar was introduced into the center of SC to become SD, as is shown in Figure 6a. We chose the width of the nanobar $W_{b}$ = 150 nm, and the length of the nanobar $L_{b}$ = 600 nm. Similarly, we plotted the *z*-component of electric field ($E_{Z}$) distributions in structure SD at the wavelength of $\lambda_{D11}$ = 1553 nm and $\lambda_{D12}$ = 1568 nm, as is shown in Figure 6b. The interference between these two antiphase modes led to the first Fano resonance at $\lambda_{D1}$ = 1556 nm.

Furthermore, we calculated the *z*-component of the magnetic field ($H_{Z}$) distributions in SD at the wavelength of $\lambda_{D21}$ = 1820 nm, $\lambda_{D22}$ = 1831 nm, respectively. Here, the black arrows in Figure 6b indicate the electric field directions, and the interference between the two antiphase modes produces the second resonance peak at $\lambda_{D2}$ = 1825 nm. Figure 6c describes how when the anapole is produced by the composition of an electric dipole and a toroid dipole, it can produce a destructive interference of their radiation patterns. For a comparison between the cases of SC and SD at *d* = 300 nm, we plotted the transmission spectra of the two metasurface structures in Figure 6d. It can be found that the missing second resonance peak reappears after the introduction of the central nanobar. Next, we will realize a dual-parameter measurement of refractive index and methane concentration based on the structure D (SD).

## 3. Measurement and Analysis

**Figure 7:**
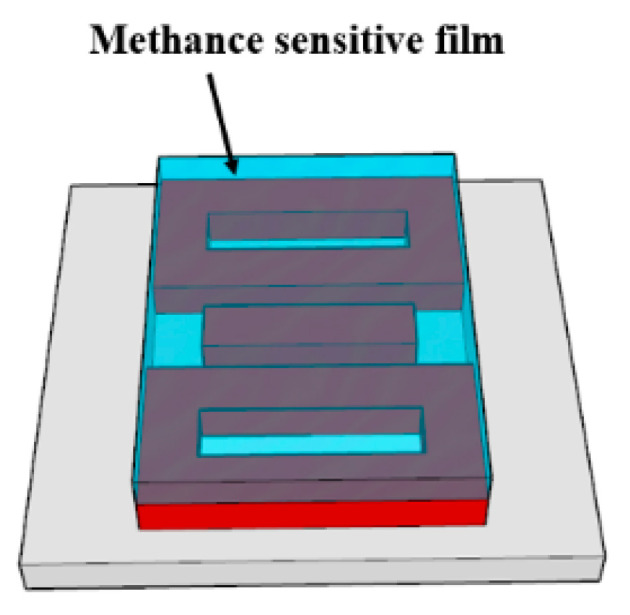
Dual-parameter sensor structure.

In this section, we will explore the sensing performance of SD. Considering that the formation mechanisms of two Fano resonances are different and the resonance peak shifts linearly with the variation of the surrounding medium, SD can be used for dual-parameter measurement. Based on above analysis results, we designed a dual-parameter sensor structure, as is shown in Figure 7. The sensor structure is coated with a methane-sensitive film with a thickness of 200 nm; the length and width of the film should cover the silicon structure exactly. We chose a methane-sensitive material UVCFS [37,38,39], which is not sensitive to temperature and humidity. Besides this, the refractive index of this methane-sensitive film decreased linearly with the increase of methane concentration within the range of 0–3%. For each 1% increase in methane concentration, the refractive index of the methane-sensitive film decreased by 0.0038 within the range of 1.4478–1.4364, as is shown in Equation (11). Since the dispersion of this material is relatively small, we can ignore the influence of dispersion on the refractive index of the material in the simulation calculations, and only consider the influence of gas concentration on the refractive index of this material for the verifications.

(11) $$N_{\mathrm{eff}}=1.4478-0.0038C_{CH_{4}}$$

**Figure 8:**
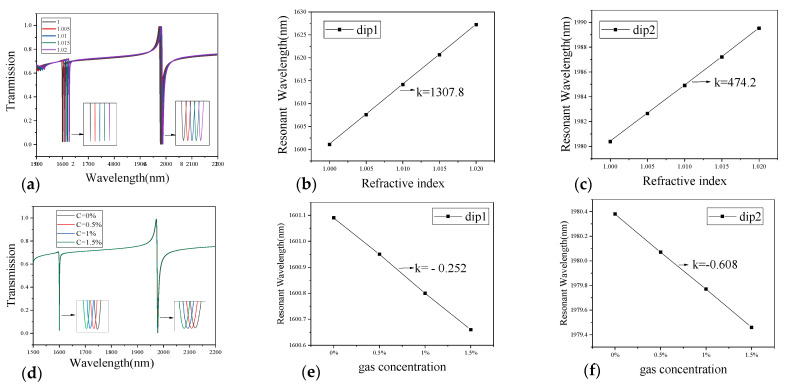
(**a**) The transmission spectra at different background indexes *n* and (**b**,**c**) the resonant wavelength shifts of dip1 and dip2 when RI changes from 1.00 to 1.02. (**d**) The transmission spectra at different gas concentrations. (**e**,**f**) the resonant wavelength shifts of dip1 and dip2 when the gas concentration varies from 0% to 1.5%.

Figure 8a,b show the transmission spectra under different values of background index (*n* = 1.00, 1.005, 1.01, 1.015, 1.02, respectively) when the gas concentration $C_{{\mathrm{CH}}_{4}}$ = 0. Both dip1 and dip2 had a red-shift with the increase of the background index. The RI sensitivities of dip1 and dip2 were 1307.8 nm/RIU and 474.2 nm/RIU, respectively. Figure 8d,e describe the transmission spectra under different gas concentrations ($C_{{\mathrm{CH}}_{4}}$ = 0%, 0.5%, 1%, 1.5%, respectively) when the background index *n* = 1.00. Here, the spectral responses of dip1 and dip2 had a blue-shift as the gas concentration rises. The gas concentration sensitivities of dip1 and dip2 were −0.252 nm/% and −0.608 nm/%, respectively.

In order to get the optimal parameters of the sensor structure, we propose an effective method to optimize the structural parameters and record the results in Table 1. To make the structure compact and easy to integrate, we keep $P_{x}=P_{y}=1500\mathrm{nm}$ and analyze the influence of three typical parameters (*t*, w, and *L*) on the gas sensitivity. It can be seen from Table 1 that the maximum sensitivity of dip1 was −0.295 nm/% at *t* = 110 nm, *w* = 500 nm, *L* = 950 nm; and the maximum sensitivity of dip2 was −0.683 nm/% at *t* = 120 nm, *w* = 550 nm, *L* = 950 nm. Since the sensitivity of dip2 was large enough, we chose *t* = 110 nm, *w* = 500 nm, *L* = 950 nm to maximize the sensitivity of dip1. Under these optimized structural parameters, we obtained the refractive index sensitivity of 1305.6 nm/RIU for dip1 and 456.6 nm/RIU for dip2, respectively.

Based on the above results, the resonant wavelength variations of the proposed sensor structure can be calculated through a matrix S, which is defined as Equation (12). In Equation (12), The matrix elements $S_{I1},S_{I2},S_{C1},S_{C2}$ represent the RI sensitivity and gas concentration sensitivity of dip1 and dip2, respectively.

(12) $$S=\left|\begin{array}{ll} S_{I1} & S_{C1}\\ S_{I2} & S_{C2} \end{array}\right|$$

The dual-parameter sensor can be expressed through the matrix S, and the resonant wavelength variations can be obtained from Equation (13), where $\Delta \lambda_{1}$ and $\Delta \lambda_{2}$ represent the resonant wavelength changes of dip1 and dip2, respectively. ΔRI and ΔC represent the changes of RI and gas concentration, respectively.

(13) $$\left[\begin{array}{l} \Delta \lambda_{1}\\ \Delta \lambda_{2} \end{array}\right]=S\cdot \left[\begin{array}{l} \Delta RI\\ \Delta C \end{array}\right]$$

Therefore, the variations of RI and gas concentration can be calculated by Equation (14).

(14) $$\left[\begin{array}{c} \Delta RI\\ \Delta C \end{array}\right]=S^{-1}\cdot \left[\begin{array}{l} \Delta \lambda_{1}\\ \Delta \lambda_{2} \end{array}\right]$$

Then, the matrix coefficients should be brought in as is shown in Equation (15).

(15) $$\left[\begin{array}{c} \Delta RI\\ \Delta C \end{array}\right]={\left[\begin{array}{cc} 1305.6nm/RIU & -0.295nm/1\%\\ 456.6nm/RIU & -0.61nm/1\% \end{array}\right]}^{-1}\cdot \left[\begin{array}{l} \Delta \lambda_{1}\\ \Delta \lambda_{2} \end{array}\right]$$

In order to discuss the dual-parameter sensor in detail, we provide the simultaneous measurement results of RI and gas concentration. As is shown in Table 2, $\Delta RI_{SET}$ and $C_{SET}$ represent the changes of presupposed RI and gas concentration, while $\Delta RI_{CAL}$ and $\Delta C_{CAL}$ represent the changes of calculated RI and gas concentration, which can be calculated by demodulation matrix Equation (13). We selected three different sets of ($\Delta RI_{SET}$, $C_{SET}$) (a (0.005,1.5%), b (0.005,2%), and c (0.015,3%)) to verify the results calculated by the demodulation matrix, and the results are listed in Table 2. The errors between the calculated value and the set value are very small and can be neglected. In other words, the proposed model structure is quite accurate.

## 4. Conclusions

Four different metasurface structures labeled as SA–SD are proposed for comparison in this paper. Both SA and SB have only one Fano resonance to realize single-parameter measurement. In order to achieve dual-parameter measurement, we designed a simple two-toroidal structure—SC—to produce two different resonance peaks in the transmission spectrum. One resonance is excited by the coupling of the electric dipole, and the other is excited by the coupling of the toroidal dipole. However, the second resonance peak will disappear if the distance between two toroidal frames is too long. That is because the coupling strength between the toroidal dipoles is too weak. To solve this problem, a nanobar was introduced into the center of SC to enhance the coupling strength, and it became the final structure—SD. Through the combined use of gas-sensitive film, we implemented a simultaneous measurement of refractive index and gas concentration based on the Fano resonance effect. The proposed sensor has the advantages of simple structure, good stability, and high sensitivity, which is beneficial for the online monitoring of multiple environmental parameters.

## Figures and Tables

**Table 1:** Parameter optimization results.

*t*/nm	*W*/nm	*L*/nm	Sensitivity/nm/%	
			Dip1	Dip2
110	450	850	−0.231	−0.52
		900	−0.29	−0.527
		950	−0.252	−0.608
	500	850	−0.252	−0.54
		900	−0.291	−0.55
		950	−0.295	−0.61
	550	850	−0.252	−0.54
		900	−0.292	−0.663
		950	−0.29	−0.667
120	450	850	−0.248	−0.53
		900	−0.252	1.456
		950	−0.291	−0.54
	500	850	−0.234	−0.55
		900	−0.291	−0.56
		950	−0.292	−0.679
	550	850	−0.237	−0.576
		900	−0.236	−0.59
		950	−0.292	−0.683
130	450	850	−0.252	−0.54
		900	−0.234	−0.55
		950	−0.255	−0.553
	500	850	−0.252	−0.563
		900	−0.237	−0.576
		950	−0.256	−0.58
	550	850	−0.233	−0.593
		900	−0.237	−0.604
		950	−0.294	−0.61

**Table 2:** The calculated results.

Sampling Points	$\Delta RI_{set}$	$\Delta C_{set}$	$\Delta \lambda_{1}$	$\Delta \lambda_{2}$	$\Delta RI_{cal}$	$\Delta C_{cal}$
a	0.005	1.5%	6.06	1.36	0.005	1.5%
b	0.005	2%	5.92	1.05	0.005	2%
c	0.015	3%	18.67	5	0.015	3.01%

## Data Availability

Not applicable.
